# Skeletal Muscle O_2_ Diffusion and the Limitation of Aerobic Capacity in Heart Failure: A Clarification

**DOI:** 10.3389/fcvm.2019.00078

**Published:** 2019-06-12

**Authors:** David Montero, Candela Diaz-Canestro

**Affiliations:** Faculty of Kinesiology, Libin Cardiovascular Institute of Alberta, University of Calgary, Calgary, AB, Canada

**Keywords:** exercise intolerance, oxygen delivery, muscle diffusion, blood flow distribution, heart failure

## Introduction

The capacity to convey oxygen (O_2_) from the atmosphere in to mitochondria essentially determines maximal aerobic metabolism in humans (1–6). The inherent constitution of the O_2_ transport and utilization chain is asymmetrical, not all steps have the same importance (7, 8). Intracellular biochemical mechanisms that could in theory limit O_2_ utilization are overbuilt in relation to the potential delivery of O_2_ through the circulatory system (2, 3, 9). Peak oxygen consumption (VO_2peak_), a hallmark of aerobic capacity elicited by incremental exercise involving more than half of total muscle mass, is mainly determined by the circulatory capacity to deliver O_2_ to working muscle even in the presence of compromised muscle oxidative capacity (5, 7, 8). Glaring evidence of the impact of the circulatory system on VO_2peak_ includes conditions such as heart failure (HF), intrinsically linked with impaired cardiac output and thus limited convective O_2_ delivery (2, 6). VO_2peak_ is a strong and independent predictor of survival in HF patients used to determine eligibility for cardiac transplantation (6, 10, 11). After diagnosis of HF, survival estimates do not exceed 50% at 5 years (12, 13).

Understanding the physiology of O_2_ delivery and thereby VO_2peak_ in HF may facilitate the identification of target mechanisms and the advent of effective treatments. While classic empirical studies in HF patients support the primary role of impaired cardiac pumping capacity in the limitation of VO_2peak_ (14, 15), a recent paradigm based on theoretical assumptions attribute the main importance to skeletal muscle abnormalities in O_2_ diffusion from capillaries in to mitochondria (16). Given the radical change of rehabilitation programs implicit in the “skeletal muscle” paradigm, herein we sought to shed light on the foundation of this relatively new tenet in the HF field. In particular, a fundamental aspect will be clarified: the measurement and calculation of O_2_ diffusion in skeletal muscle.

## O_2_ Transport Assessment: de facto Measurement of O_2_ Diffusion in Skeletal Muscle

The transport of O_2_ in living organisms follows well-known physical phenomena. O_2_ molecules move via (i) convection, due to the bulk motion of fluids, and (ii) diffusion, spontaneously spreading out from a region of high concentration to a region of low concentration. Along the O_2_ cascade, convection is the mode of O_2_ transport between the atmosphere and the lungs, and between pulmonary capillary blood and tissue microvascular beds, respectively determined by the bulk motion of air and circulating blood. Diffusion of O_2_ mainly occurs from alveoli in to pulmonary capillaries, and from tissue microvascular beds in to mitochondria. With respect to the measurement of O_2_ transport, both convection steps (air-to-lung, blood circulation) can be measured with relatively high accuracy in humans by means of spirometers, air/blood gas analyzers, arterial/venous blood samples and indicator-dilution/ultrasound techniques (17, 18). These well-established research methods may also be used to assess lung O_2_ diffusion. The final O_2_ diffusion step in the skeletal muscle microcirculation, however, cannot yet be directly measured. The level of resolution required to capture O_2_ extraction in microvessels supplying active muscle fibers is beyond reach owing to technical limitations including temporal and spatial constraints (8, 19).

The possibility seemingly exists, nonetheless, to make use of partial measurements and multiple assumptions to deliver a quantitative value for skeletal muscle O_2_ diffusion capacity (DMO_2_) (20–22). Notably, in the field of HF (16), some clinical researchers are currently applying a method for estimating DMO_2_ conceived almost 3 decades ago (20–22). Herein, DMO_2_ is portrayed as the ratio of skeletal muscle O_2_ consumption (VO_2_) and O_2_ pressure gradient between microvessels and mitochondria (21, 23, 24).

$$\begin{array}{l} {\text{DMO}}_{2}\;=\;\frac{\text{skeletal}\;\text{muscle}\;{\text{VO}}_{2}}{{\text{O}}_{2}\;\text{pressure}\;\text{gradient}} \end{array}$$

**Figure 1:**
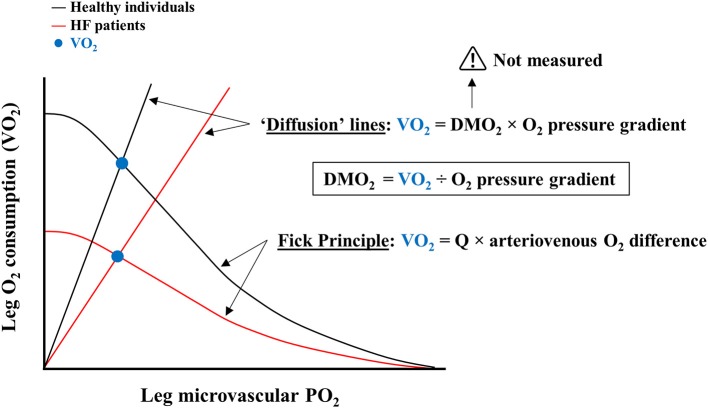
Common schematic illustration of convective and diffusive components of oxygen consumption (VO_2_) by proponents of abnormalities in skeletal muscle O_2_ diffusion. Leg O_2_ consumption is represented as a function of leg microvascular partial pressure of oxygen (PO_2_). Black lines depict Fick and “Diffusion” lines for healthy control individuals; red lines refer to heart failure (HF) patients. The intersection of these lines reflects the VO_2_ achieved (blue circles). Curved lines are derived from the established Fick Principle. Straight “Diffusion” lines are calculated by the product of skeletal muscle O_2_ diffusion capacity (DMO_2_) and O_2_ pressure gradient. Given that DMO_2_ cannot be directly measured, the “Diffusion” lines are intrinsically dependent on VO_2_, which is at least in part determined by cardiac output (Q), i.e., convective O_2_ delivery, according to the Fick Principle. Hence, “Diffusion” lines are compounded representations of convective and diffusive components of VO_2_.

At first sight the notion of DMO_2_ appears consistent, albeit a close scrutiny of the actual measurements reveals salient incongruences. Skeletal muscle O_2_ consumption—calculated by the product of leg blood flow (LBF) and the difference between femoral arterial and venous O_2_ content (16, 21)—is primarily determined by convective O_2_ delivery, since LBF is substantially impaired (up to −40%) in HF conforming to the reduced pumping capacity of the failing heart (6, 25). Moreover, femoral vein O_2_ content is close to zero in HF patients at VO_2peak_ (2). Therefore, the first component (numerator) of the DMO_2_ equation, i.e., skeletal muscle VO_2_, is essentially a function of convective O_2_ delivery (LBF × arterial O_2_ content) a fundamental mathematical flaw for a variable claimed to represent diffusive O_2_ transport (Figure 1).

The O_2_ pressure gradient between skeletal muscle microvessels and mitochondria is also estimated from femoral arterial and venous O_2_ content measurements, both pertaining to the macro- instead of microcirculation (16, 21). A myriad of assumptions are thus necessary to infer the postulated denominator of the DMO_2_ equation (20). For instance, the inherent heterogeneity of leg microvascular blood flow (26, 27), which even at VO_2peak_ perfuses tissues (e.g., adipose tissue, bone, inactive muscle) not demanding a high VO_2_, is neglected (20, 21). Similarly, altered capillarization as well as anatomical and/or functional shunting within the lower limb, which may have a substantial influence in HF patients at VO_2peak_ (28, 29), is ignored (16, 21). Taken together, the estimation of the O_2_ pressure gradient entails as a necessary premise that all blood flow downstream of the femoral artery perfuses active muscle fibers, in a perfect match between O_2_ delivery and metabolic demand, an untenable shortcoming (26–28).

Considering the actual measurements underpinning the concept of DMO_2_, its mathematical equation would be more accurately expressed as:

$$\begin{array}{l} {\text{DMO}}_{2}\;=\;\frac{\text{LBF}\;\times \;\text{arteriovenous}\;{\text{O}}_{2}\;\text{difference}\;}{\text{Blood}\;\text{flow}\;\text{distribution}\;\times \;{\text{O}}_{2}\;\text{pressure}\;\text{gradient}} \end{array}$$

Hence, the numerator and denominator of DMO_2_ comprise variables reflecting convective O_2_ delivery, LBF and blood flow distribution, respectively. The observation of reduced DMO_2_ in HF patients is therefore not surprising (16, 23, 24). To conclude from these studies that mechanisms underlying skeletal muscle O_2_ diffusion should be primarily targeted for therapy is questionable (30). Caution should be taken in the interpretation of lower DMO_2_ in HF patients, which can be largely attributed to abnormalities in convective O_2_ delivery, let alone presenting DMO_2_ results as the main buttress of a new paradigm (31, 32). Further research taking advantage of technological developments in measurement accuracy and resolution of O_2_ dynamics in skeletal muscle will have to elucidate its role in the limitation of VO_2peak_ in HF populations.

## Author Contributions

DM and CD-C contributed to study design, interpretation, and manuscript writing.

### Conflict of Interest Statement

The authors declare that the research was conducted in the absence of any commercial or financial relationships that could be construed as a potential conflict of interest.
